# Continuum of Care UNAIDS Fast-Track Targets Evaluation of Patients Living with Human Immunodeficiency Virus Infection

**DOI:** 10.3390/healthcare9101249

**Published:** 2021-09-23

**Authors:** Cristian Jianu, Corina Itu-Mureşan, Adriana Violeta Topan, Irina Filipescu, Mihaela Elena Jianu, Carmen Stanca Melincovici, Carmen Mihaela Mihu, Sorana D. Bolboacă

**Affiliations:** 1 Department of Medical Informatics and Biostatistics, “Iuliu Hațieganu” University of Medicine and Pharmacy, 400349 Cluj-Napoca, Romania; cristianjianu1@gmail.com (C.J.); sbolboaca@umfcluj.ro (S.D.B.); 2 Department of Immunosuppressed, Clinical Hospital of Infectious Diseases, 400348 Cluj-Napoca, Romania; corinaitu@yahoo.com (C.I.-M.); topanadriana@yahoo.com (A.V.T.); irinukfilipescu@gmail.com (I.F.); 3 Department of Infectious Diseases, “Iuliu Hațieganu” University of Medicine and Pharmacy, 400348 Cluj-Napoca, Romania; 4 Department of Histology, “Iuliu Hațieganu” University of Medicine and Pharmacy, 400349 Cluj-Napoca, Romania; carmen.melincovici@umfcluj.ro (C.S.M.); carmenmihu@umfcluj.ro (C.M.M.)

**Keywords:** human immunodeficiency virus (HIV) infection, Fast-Track targets (FTTs), antiretroviral therapy (ART), HIV viral load, continuum of care, sexual behavior, epidemiology

## Abstract

The current study evaluated the progress of continuum healthcare for patients living with human immunodeficiency virus (HIV) infection from Cluj County in two moments, 2016 and 2020, and compared the results to the Fast-Track targets (FTTs) proposed by the Joint United Nations Programme (UNAIDS) on HIV/AIDS. By the end of 2020, 368 out of 385 confirmed HIV-positive patients from Cluj County were under surveillance in our center, representing almost 95% of the patients living with HIV and knowing their diagnosis, compared to 87.9% in 2016. Nearly 97% of those in active follow-up from Cluj County were under antiretroviral therapy (ART) in 2020, compared to 89% in 2016. The number of virally suppressed patients from those under ART was almost 94% in 2020, compared to 82.7% in 2016, and the increase is observed regardless of the ART regime. A shift towards integrase strand transfer inhibitors, with a higher efficacy, fewer adverse effects, and fewer drug interactions, is observed, which could contribute to the decrease in HIV transmission.

## 1. Introduction

In the last thirty years, infection with human immunodeficiency virus (HIV) has spread worldwide, and still raises several challenging issues for the general health status of the population and the welfare of the economic and healthcare systems [1,2]. Worldwide, at the end of 2019, 38 million people were already diagnosed and living with HIV, 67% of them had access to antiretroviral therapy, and allegedly 7.1 million people were not yet diagnosed (living with HIV but did not get tested and do not know that they have the infection) [3]. On 30 June 2020, 16,658 patients were living with HIV in Romania [4].

The Joint United Nations Programme (UNAIDS) proposed in 2014 the following Fast-Track targets (FTTs) for 2020: 90% of patients living with HIV to know that they are infected, 90% of those infected to be on ART (antiretroviral therapy), and 90% of patients on ART to have an undetectable viral load [5,6]. When these 90-90-90 targets are achieved, the percentage of all patients virally suppressed is estimated to be 73% of all people living with HIV [5]. Furthermore, UNAIDS, in 2014, estimated that the world AIDS (acquired immunodeficiency syndrome) epidemic would end by 2030 if the proposed targets are achieved by 2020 [5]. The guidelines for HIV treatment changed in 2015, recommending starting antiretroviral therapy regardless of the number of CD4 T lymphocytes [7]. Until 2015, only patients with CD4 counts below 500/mm^3^ had a recommendation for antiretroviral therapy [8]. The treatment guidelines have also been changed over time, and a new class of drugs, namely integrase strand transfer inhibitors—INSTIs (e.g., raltegravir, dolutegravir, elvitegravir), have been introduced in the standard treatment. Due to their higher efficacy, fewer adverse effects, and fewer drug–drug interactions, INSTIs became the first line of antiretroviral therapy [6,9]. Elvitegravir (EVG), dolutegravir (DTG), and raltegravir (RAL) proved an efficiency with a viral load suppression ≥ 90% (viral load < 50 copies/mL) in HIV-infected treatment-naive and -experienced patients who followed an INSTI-based cART-regimen [10]. Kolakowska et al. [11] reported a higher antiviral potency of INSTIs compared to other classes of ART. However, contradictory neuro-psychiatric effects, especially for DTG, weight gain for DTG, lower adverse effects rates of bictegravir (BIC), and the caution of elvitegravir/cobicistat in patients with other co-morbidities are reported [11].

Limited results were published in the scientific literature to date regarding UNAIDS FTTs’ achievements in countries worldwide. Levi et al. conducted a systematic review of continuum care in 196 countries, evaluated 69 countries [12], and concluded that none of the evaluated countries met the 90 (up to 87% in the Netherlands)-90 (up to 71% in Switzerland)-90 (up to 68% in Switzerland) targets. Huerga et al. [13] reported higher values of 90-90-90 targets on women (79%-71%-93%) compared to men (68%-68%-92%) on the South Africa population from Mbongolwane, Eshowe, and KwaZulu-Natal provinces. The reported progress towards 90-90-90 on the Kediri city population in Indonesia was 6.4%-74.9%-9.9%, as reported by Sekalembe et al. in 2020 [14]. In Rwanda, a study conducted in 2018 reported 97% of people living with HIV on ART, and 91% of those on ART were virally suppressed [15]. The 90-90-90 targets reported in 2018 by Brown et al. showed the highest percentages in the West European countries (87%-91%-93%), lowest values in the East European countries (76%-46%-78%), and intermediate values in Central European countries (83%-73%-75%) [16]. In Romania, the reported HIV prevalence is 0.07%, with an annual incidence of 0.002%, and 850 cases/year on average from 2011 to 2017 [17]. A decrease in AIDS incidence and mortality due to ART and a reduction in pediatric HIV infection is observed in Romania [18]. The Romanian cohort, a particularity of this country, is formed by patients younger than 13 years infected iatrogenically before 1991 with HIV, in most cases in hospitals and orphanages [19]. Limited information is available in the scientific literature regarding the 90-90-90 FTTs achievement in Romania. The European Centre for Disease Prevention and Control (ECDC) reported in May 2017 the following data for Romania: 98% of people living with HIV were diagnosed, 75% were under ART, and 38% had viral suppression [20]. We aimed to evaluate the UNAIDS 90-90-90 Fast-Track targets’ achievements by comparing the continuum-of-care data of HIV-infected patients living in Cluj County in two moments: the end of 2016 and 2020.

## 2. Materials and Methods

### 2.1. Study Design

We have conducted a retrospective cross-sectional analysis of routine clinical data from medical charts of patients living with HIV infection (PLHIV) with a domicile in Cluj County and who were under care at the time of analysis at Cluj-Napoca AIDS Center, Romania. The Cluj-Napoca AIDS Center mainly monitors HIV-infected patients from North-West Romania (Cluj, Bihor, Maramureş, Satu Mare, and Sălaj counties). In Romania, PLHIV freely received the ART(s) at the level of the county where they reside, and the Cluj-Napoca AIDS Center had no control over ART(s) release. For this reason, only PLHIV with a domicile in Cluj Country (could verify the release of ART(s) medication) accomplished the inclusion criteria evaluated, and no sampling method was applied.

The study was conducted according to the principles of the Helsinki Declaration and was approved by the Ethical Committee of the Iuliu Hațieganu University of Medicine and Pharmacy Cluj-Napoca (approval no. 144 from 4 February 2018) with waived patient consent.

### 2.2. Clinical Procedures, Study Population, Outcomes, and Other Variables

The HIV diagnosis was made following the case definition of the Centers for Disease Control and Prevention 1993 [21] and the guidelines of the European Center for Disease Control [22]. In Romania, ART(s) treatment is freely available as a part of the National Program for Monitoring and Surveillance of HIV/AIDS Infection [23].

We included in this analysis all PLHIV:

(1) regardless of age and gender;
(2) enrolled in care at the Cluj-Napoca AIDS Center, Romania in December 2016 and, respectively, December 2020;
(3) with domicile in Cluj County;
(4) and having complete clinical routine data regarding diagnosis, ART(s) regimen, and/or viral load.

Patients with HIV infection with a domicile in other counties but under care in our center were excluded from this study due to the absence of access to ART(s) release. All HIV-infected subjects in care in December 2016 and, respectively, 2020 were evaluated regardless of the time since they were included in the care at Cluj-Napoca AIDS Center. The 2020 year was particular, due to the fact that the COVID-19 pandemic restricted access to hospitals, so the raw data of PLHIV were collected from the latest clinical and paraclinical evaluation carried out in 2019 whenever no evaluation exists in 2020. The raw data for eligible patients were extracted from the clinical database on 10 March 2021.

Demographic (e.g., gender, age in 2016 and, respectively, 2020, disease duration), way of infection (e.g., heterosexual, men sex with men (MSM), mother-to-child, etc.), and routine clinical data (e.g., CD4/mm^3^, ART(s), and viral load) were retrieved from medical charts of the eligible PLHIV, who accomplished the inclusion criteria as closest as possible to the end of December 2016 and, respectively, December 2020.

### 2.3. Statistical Analysis

The following definitions were used in this study: *on ART* (defined as having an available ART initiation date and being on ART at the end of 2016 and, respectively, 2020), *viral load* (viral load measured as closely as possible to the date of interest, end of 2016 and, respectively, end of 2020), and *virally suppressed* (most closed viral load to December 2016 and, respectively, 2020 with viral load < 200 copies of HIV/mL). The age groups used in reporting results follow the WHO guideline [24]: adult (age > 19 years), adolescent (10 ≤ age ≤ 19 years), and child (1 ≤ age < 10 years).

90-90-90 targets were calculated as a percentage based on the raw data according to the 2018 UNAIDS Global AIDS Monitoring indicators. However, the percentage of all PLHIV who know their status is just an estimation calculated as the number of PLHIV in active surveillance out of the total number of confirmed HIV-positive persons with a domicile in Cluj County, since no populational HIV testing was applied. The frequency rate in 2020 was calculated, considering that the population of Cluj County on January 1st, 2020 was 737.992 persons, according to the Cluj County Directorate of Statistics.

The percentage of Cluj County HIV-infected patients in active surveillance was reported by referring to the confirmed HIV-infected person with a domicile in the Cluj County for each evaluated year. The gender and way of HIV infection under active follow-up were reported as the number and associated percentage. The FTT components were reported as a percentage, and the values were compared to the target value (namely 90%) using one proportion Z-test [25]. The Chi-squared test or Fisher’s exact test was used to compare the frequencies regarding different ART regimes. The exploratory analysis was carried out at a significance level of 5%, and the *p*-values less than 0.05 were considered statistically significant. 

## 3. Results

In December 2016, we had 467 PLHIV from different counties in active follow-up at the Cluj-Napoca AIDS Center, and the number increased to 738 PLHIV in December 2020. Two hundred and fifty-four PLHIV (54.4%) had a domicile in Cluj County in December 2016, and the number increased to 368 PLHIV (49.9%) in December 2020. In 2016, 289 confirmed HIV-infected patients were living in Cluj County, and 254 of them were under active follow-up (87.9%, *p*-value = 0.2622, as compared to the established target of 90%—one proportion Z-test). At the end of 2020, 385 confirmed HIV-patients were living in Cluj County, and 368 were under active follow-up (95.6%, *p*-value = 0.0004—one proportion Z-test).

Most of the evaluated patients were men, and heterosexual transmission of HIV infection was the most common in our sample (Table 1). 

Almost 89% of HIV-infected patients living in Cluj County in December 2016 were under ART. The cohort evaluated in December 2016 was predominantly formed by adults (218, 96.5–82.4% virally suppressed), with five adolescents (2.2%, all virally suppressed with undetectable viral load) and three children (1.3%, all virally suppressed with undetectable viral load). Similarly, at the end of December 2020, the evaluated cohort comprised adults (361, 98.1–91.7% virally suppressed), with six adolescents (1.6–83.3% virally suppressed; undetectable viral load with one exception) and one child (0.3%, virally suppressed with undetectable viral load). In the evaluation conducted in 2016, 131 patients (57.96%) had CD4 > 500/mm^3^ and 15 (6.64%) had CD4 < 200/mm^3^. In the evaluation carried out in December 2020, the number of patients with CD4 > 500/mm^3^ increases (261, 70.9%), whereas the number of patients with CD4 < 200/mm^3^ decreases (14, 3.1%).

Twenty-one new HIV cases were reported in 2020, with a frequency of 2.85 to 100.000 persons in Cluj County. The number of HIV patients living in Cluj County under healthcare increased by 114 at the end of 2020 compared to 2016, with significantly more patients under ART in 2020 (Table 2). The viral load of 35 HIV-infected subjects had their measurements collected in 2019.

The percentage of all people living with HIV infection who were virally suppressed (viral load undetectable or viral load between 50 and 200 copies/mL) was 81.9% (*p*-value < 0.0001, as compared to the theoretical value of 90%) in December 2016, and was raised to 91.6% in December 2020 (*p*-value = 0.3135, as compared to the theoretical value of 90%).

Two hundred and four patients were in both evaluated cohorts (2016 and 2020), and 146 patients with an undetectable viral load in 2016 remained with the same status in 2020, whereas 31 with a viral load ≥ 50 copies/mL in 2016 became undetectable in 2020.

A change in the antiretroviral therapy is seen in 2020 as compared to 2016, with fewer patients on PI and NNRTIs (non-nucleoside reverse transcriptase inhibitors), but more patients on INSTIs (Table 3).

**Figure 1:**
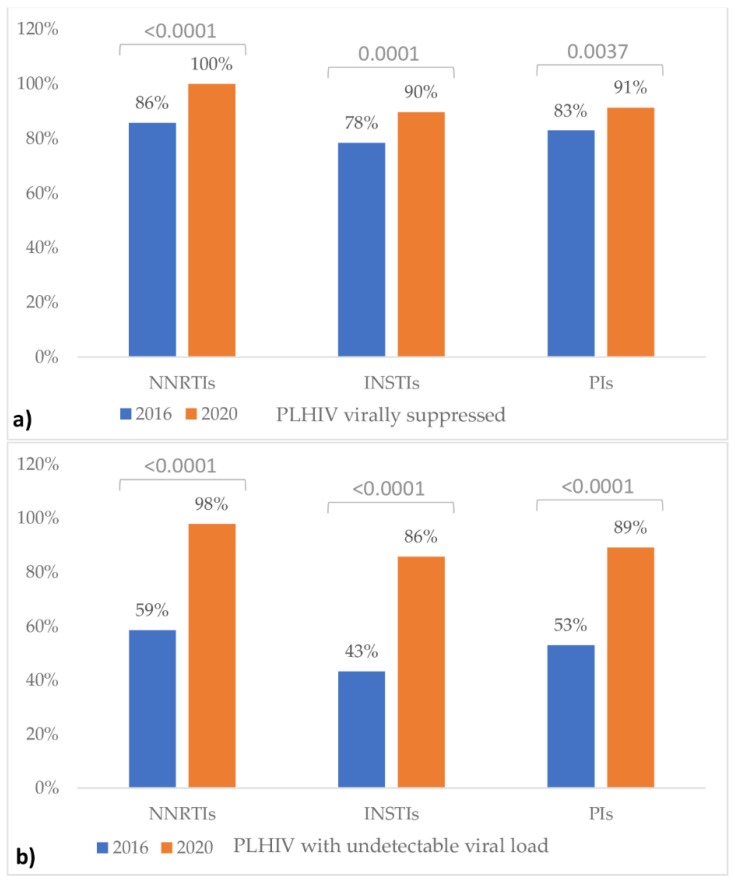
Percentage of PLHIV with viral suppression (**a**) and, respectively, PLHIV with undetectable viral load (**b**) by ART regime. PLHIV = patients living with HIV infection; ART = antiretroviral therapy; NNRTIs = non-nucleoside reverse transcriptase inhibitors; NSTIs = integrase strand transfer inhibitors; PIs = protease inhibitors. The *p*-values above the columns correspond to the Z-test for proportions.

Neither the viral suppression nor the undetectable viral load proved significantly associated with the ART regime (*p*-values > 0.05). A significant increase in the percentage of PLHIV virally suppressed and, respectively, PLHIV with an undetectable viral load, was observed in 2020 compared to 2016 (Figure 1).

## 4. Discussion

Our exploratory analysis was conducted on patients from Cluj County living with HIV infection in December of 2016 and December of 2020 in order to estimate that the meeting of Fast-Track targets (FTT) of the World Health Organization, namely 90-90-90, was partially successfully done. In 2020, 95.6% of HIV-positive people living in Cluj County were active in follow-up, and thus both had their status known and accepted the disease, 97.3% from those with active follow-up were under ART, and 91.6% achieved viral suppression. However, we did not successfully evaluate the percentage of all people living with HIV who know their HIV status, since we collected the data from medical charts and no Cluj County populational HIV screening was conducted. 

The observed frequency of new HIV cases in 2020 in our sample (2.85/100.000) is similar to other worldwide reported values, as 2.32–2.72 to 100.000 persons [4,23,26].

At the end of 2016, in Romania, 83% of the estimated total number of people living with HIV (PLHIV) have been diagnosed [27]. In 2018, 88% of the estimated number of people living with HIV from our country had been diagnosed [28], which is above the Central Europe regional average of 83% [16]. The increase in the HIV patients under surveillance reported to the number of confirmed infections (Table 2) in 2020 compared to 2016 showed a percentage higher than 90%. However, this result reflects only the number of HIV-infected patients under surveillance reported to the confirmed HIV-infected patients, while patients who do not know they are HIV-infected certainly exist. Furthermore, people who know that they are infected but are not in an active follow-up at our center also exist.

Our exploratory analysis showed a predominance of men in the evaluated cohort (Table 1), following the pattern reported by ECDC and the WHO Regional Office for Europe (HIV/AIDS surveillance in Europe 2019–2018 data) [29]. This finding showed the predominance of the disease among men. Still, the lower frequency of women could also be explained by women’s social stigma regarding sexually transmitted diseases and thus the tendency to postpone medical evaluation, leading to an advanced stage diagnosis of the HIV infection [28] and, therefore, a less satisfactory treatment outcome.

The upward trend in the transmission of HIV infection by MSM has been maintained in our population in the last four years (Table 1) and follows the same tendency since the beginning of HIV infection surveillance in our center [30]. The men who have sex with men transmission of HIV infection has been reported to increase in Central and Western Europe [29]. A socio-cultural open-minded view could explain the observed MSM increase towards sexual behaviorism in the context of the scientific, medical, and industrial development of North-West Romania.

The increase in HIV-infected patients under antiretroviral therapy by 8.3% in 2020 compared to 2016 showed the achievement of the second target recommended by UNAIDS (Table 2). The percentage in Cluj County was above our country average of 91.30% last reported in June 2020 [4], and above the 73% for Central Europe reported at the end of 2018 [16]. A careful evaluation of HIV-infected patients and an increase in the patient’s knowledge of controlling the disease progression could explain the higher adherence of the patient to the treatment and a higher number of patients under active surveillance, and, thus, the optimal treatment.

The number of patients with HIV infection and under ART with a viral load below 200 copies/mL has increased from 81.9% (2016) to 91.6% (2020), achieving the third target proposed by UNAIDS for 2020; this increase was observed regardless of the ART regime (Figure 1). The patients with an HIV viral load under 200 copies/mL are considered to have a low risk of transmission of HIV infection [31], so this target contributes to the decrease in HIV transmission. In our study, the percentage of patients with an HIV viral load below 50 copies/mL (undetectable, Table 2) increased by 15.9% at the end of 2020 compared to 2016. In Romania, the patients with an HIV viral load below 200 copies/mL is 81.2%, and those with an undetectable viral load is 73.2% [27]. Our results showed that HIV-infected subjects who live in Cluj County are virally suppressed at a higher percentage than the nationally reported data (Table 2 and Figure 1). Furthermore, despite the epidemiologic condition in 2020, the active surveillance of HIV-infected subjects living in Cluj County regarding the viral load was higher than the one reported in 2019 in Romania (78.60%) [32]. In Central Europe, the percentage of patients under ART who are virally suppressed is 75%, and, from all patients with HIV infection, the percentage is 46% [16]. In Cluj County, the data are above the regional averages (Table 4) [4,33].

The results reported in our study could be explained by the fact that ART is initiated within seven days after the diagnosis, and the adherence to the treatment is high. We have generous support from the psychologist in evaluating the patients, increasing their commitment to therapy. In addition, the efficacy of the newest drugs used is another factor that could explain our results. When considering different antiretroviral regimens, we can observe that the number of patients treated with INSTIs has increased from 17.3% to 43.6% in our county (Table 3). These drugs are currently the preferred first-line used treatment regimen worldwide [34,35] due to their high efficacy and fewer adverse effects and drug interactions. In our HIV center, we comply with the latest guidelines in HIV treatment and disease monitoring, and over the past four years, we have obtained good results using INSTIs as recommended by the European AIDS Clinical Society (EACS). The number of patients in our study undergoing the PI regimen has significantly decreased (Table 3) because the treatment schedule involves the intake of many tablets per day, with adverse effects that are more frequent than other ARTs [36]. The number of patients treated with an NNRTI regimen also significantly decreased in 2020 (Table 3). These drugs proved effective in the viral load reduction (Figure 1) and currently remain an alternative therapeutic regimen, since they have a high rate of side effects. The risk of viral resistance increases if the correct administration of doses according to the schedule is omitted [37].

We have limited patients with single tablet regimen (2020, Table 3) because these drugs have only become available through the national treatment program for HIV-infected people in Romania since 2017. The comparison presented in Table 3 regarding the single tablet regimen must be carefully interpreted, considering its availability since 2017. 

Our exploratory study showed that the “90” FTTs (the first FTT is an estimation) proposed by the UNAIDS for 2020 was reached in December 2020, with results above the national media and similar to Central European values. This outcome supports the conclusion that the continuum of care in the Cluj-Napoca AIDS Center is at international standards, in line with the UNAIDS FTTs of ending the AIDS epidemic by 2030. However, a Cluj County populational study is needed to confirm the achievement of the first 90 FTT.

### Study Limitations

Our study has several limitations. First, even though we have patients under surveillance from different counties in our center, our investigated sample was narrow because ART is released only to those who had a residence in Cluj County; thus, we have this information only for them. Consequently, the results strictly reflect the investigated cohort, and the generalizability could not be appropriate. The evaluation of a cohort from only one county could be seen as irrelevant in an international context. However, knowing that the targets of continuum care are achieved at our AIDS Center could be helpful in the context of cross-border health care in the European Union and/or decisions regarding choosing where to study or live. Second, due to sample composition, no age-stratified analysis was appropriate, as recommended in the March 2021 WHO guideline [23]. Third, data collection from medical charts made the analysis being able to consider the confounding variables impossible. Such research would be helpful to identify the groups that require more attention and to define specific care paths to improve their health. 

## 5. Conclusions

The frequency of HIV infection in Cluj County is similar to data reported in other HIV surveillance centers in Romania. Almost 96% of people in Cluj County with HIV-infection know their HIV status (are aware of the disease and are under medical surveillance). Furthermore, 97% of them are under antiretroviral treatment, and nearly 94% have achieved viral suppression. Regardless of the ART regime, viral suppression significantly increased in 2020 compared to 2016. The current antiretroviral regimens are switched to INSTIs with a higher efficacy, fewer adverse effects, and fewer drug interactions that would decrease HIV transmission.

## Figures and Tables

**Table 1:** Main characteristics of the evaluated patients by year.

Characteristic	December 2016 (*n* = 254)	December 2020 (*n* = 368)
Gender, no. (%) Men Women	182 (71.7) 72 (28.3)	280 (76.1) 88 (23.9)
Way of HIV infection, no. (%) Heterosexual MSM Romanian cohort Parenteral not IDU Mother to child IDU	147 (57.9) 79 (31.1) 21 (8.3) 4 (1.6) 5 (2) 1 (0.4)	194 (52.7) 138 (37.5) 23 (6.3) 5 (1.4) 5 (1.4) 3 (0.8)

Data are expressed as numbers (percentage); MSM = men who have sex with men; IDU = intravenous drug users; Romanian cohort = children infected iatrogenically before 1991 with HIV, in most cases in hospitals and orphanages [19].

**Table 2:** HIV patients: demographics and viral load.

Patients under ART	December 2016 (*n* = 254)	December 2020 (*n* = 368)
No. (%)	226 (88.98) ^a^	358 (97.28) ^b^
Age, years	36 (29 to 48) {7 to 75}	38 (32 to 47) {8 to 79}
Men	168 (74.3)	280 (76.1)
Living in rural areas	56 (24.8)	95 (25.8)
Disease duration, years	5 (2 to 9) {0 to 23} ^#^	6 (2 to 10) {0 to 27}
CD4 count/mm^3^	562 (384 to 789)	644 (475 to 897)
Viral load		
undetectable *	170 (75.2)	326 (91.1)
between 50 and 200 copies/mL	15 (7.5)	11 (3.1)
>200 copies/mL	38 (16.8)	31 (8.7)
without virological evaluation	2 (0.9)	0 (0.0)

Excepting age, disease duration, and CD4 count, data are reported as no. (%); age is reported as median (Q1 to Q3) {min to max}, where Q is the value of the quartile, min is the minimum, and max is the maximum value; ^#^ *n* = 224; * with viral load ≤ 50 copies/mL; ^a^ *p*-value = 0.5866 (as compared to theoretical value of 90% by one proportion Z-test); ^b^ *p*-value < 0.0001 (as compared to a theoretical value of 90% by one proportion Z-test).

**Table 3:** Antiretroviral therapy regimes.

ART	December 2016 (*n* = 254)	December 2020 (*n* = 368)	Χ^2^ (*p*-Value)
Protease inhibitors (PIs)	117 (51.8)	140 (39.1)	9.0 (0.0027)
Non-nucleoside reverse transcriptase inhibitors (NNRTIs)	70 (31.0)	54 (15.1)	20.9 (<0.0001)
Integrase strand transfer inhibitors (INSTIs)	39 (17.3)	164 (45.8)	49.8 (<0.0001)
single tablet regimen	0 (0.00)	20 (5.58)	n.a. (0.2602) *

Data are reported as no. (%); Χ^2^ = statistics of the Chi-squared test; the critical X^2^ at a significance level of 5% form 2 × 2 contingency table is 3.84; n.a. = not available; * Fisher exact test.

**Table 4:** WHO’s targets for Central Europe, Romania, and Cluj County.

Region	Diagnosed	Patients on ART	Virally Suppressed	Virally Suppressed of All Patients
Target	90	90	90	73
Central Europe (2018) [31]	83	73	75	46
Romania (2019) [4]	83.16 *	91.3	81.2	58.2
Cluj County (2020)—the current study	95.58 **	97.28	94.1	91.6

Data are reported as %; * percentage calculated based on data reported in [4]; ** this percentage reflects the number of PLHIV under active surveillance reported to the total number of HIV-positive patients living in Cluj County; ART = antiretroviral therapy.

## Data Availability

Data are contained within the article.
